# Temporal/compartmental changes in viral RNA and neuronal injury in a primate model of NeuroAIDS

**DOI:** 10.1371/journal.pone.0196949

**Published:** 2018-05-11

**Authors:** R. Gilberto González, Robert Fell, Julian He, Jennifer Campbell, Tricia H. Burdo, Patrick Autissier, Lakshmanan Annamalai, Faramarz Taheri, Termara Parker, Jeffrey D. Lifson, Elkan F. Halpern, Mark Vangel, Eliezer Masliah, Susan V. Westmoreland, Kenneth C. Williams, Eva-Maria Ratai

**Affiliations:** 1 Department of Radiology, Neuroradiology Division, Athinoula A. Martinos Center for Biomedical Imaging, Massachusetts General Hospital, Charlestown, MA, United States of America; 2 Harvard Medical School, Boston, MA, United States of America; 3 Biology Department, Boston College, Chestnut Hill, MA, United States of America; 4 New England Primate Research Center, Southborough, MA, United States of America; 5 AIDS and Cancer Virus Program, Leidos Biomedical Research, Inc., Frederick National Laboratory for Cancer Research, Frederick, MD, United States of America; 6 Institute for Technology Assessment, Department of Radiology, Massachusetts General Hospital, Boston, MA, United States of America; 7 Department of Neurosciences, University of California at San Diego, La Jolla, CA, United States of America; University of Pittsburgh Centre for Vaccine Research, UNITED STATES

## Abstract

Despite the advent of highly active anti-retroviral therapy HIV-associated neurocognitive disorders (HAND) continue to be a significant problem. Furthermore, the precise pathogenesis of this neurodegeneration is still unclear. The objective of this study was to examine the relationship between infection by the simian immunodeficiency virus (SIV) and neuronal injury in the rhesus macaque using *in vivo* and *postmortem* sampling techniques. The effect of SIV infection in 23 adult rhesus macaques was investigated using an accelerated NeuroAIDS model. Disease progression was modulated either with combination anti-retroviral therapy (cART, 4 animals) or minocycline (7 animals). Twelve animals remained untreated. Viral loads were monitored in the blood and cerebral spinal fluid, as were levels of activated monocytes in the blood. Neuronal injury was monitored *in vivo* using magnetic resonance spectroscopy. Viral RNA was quantified in brain tissue of each animal *postmortem* using reverse transcription polymerase chain reaction (RT-PCR), and neuronal injury was assessed by immunohistochemistry. Without treatment, viral RNA in plasma, cerebral spinal fluid, and brain tissue appears to reach a plateau. Neuronal injury was highly correlated both to plasma viral levels and a subset of infected/activated monocytes (CD14+CD16+), which are known to traffic the virus into the brain. Treatment with either cART or minocycline decreased brain viral levels and partially reversed alterations in *in vivo* and immunohistochemical markers for neuronal injury. These findings suggest there is significant turnover of replicating virus within the brain and the severity of neuronal injury is directly related to the brain viral load.

## Introduction

HIV infection commonly results in significant neurocognitive abnormalities identified as HIV-associated neurocognitive disorders (HAND) [1–3]. While the incidence of severe neurological symptoms has been seen to decrease with highly active antiretroviral therapy (HAART), less severe versions of the disease persist among the infected population [4]. The overall prevalence of HAND and associated morbidity remain high at approximately 50% [5–7]. Major hurdles to the development of effective HAND treatments are 1) an incomplete understanding of pathogenic pathways culminating in neuronal injury and 2) the inability to characterize temporal and cumulative features of neuronal injury.

There is a consensus that HIV enters the central nervous system (CNS) during the early stages of infection primarily through virally infected/activated monocytes from the blood across the blood-brain barrier (BBB) [8, 9]. Although the virus does not directly infect neurons, neurons suffer injury due to indirect mechanisms mediated by host proinflammatory and viral proteins [8, 10–13]. The multifactorial nature of neuronal injury confounds efforts to elucidate specific neuropathogenic pathways and challenges monotherapy approaches [14, 15].

In addition to the complexity presented by multiple potential pathways to neuronal injury, little is known about the temporal process of neuronal injury itself. Reversible neuronal injury has been demonstrated at both structural and metabolic levels [16–18], and improvement in neuropsychological performance has been observed up to three years after introduction of HAART in some individuals [19, 20]. The lack of clear demarcation of reversible and non-reversible components of neuronal injury may confound treatment studies [16] and be an important consideration in interpreting the varying degrees of HAART’s effectiveness in ameliorating HAND progression.

The simian immunodeficiency virus (SIV)-infected rhesus macaque shares very similar pathology with HIV-infected human patients, including the development of AIDS, disease of the CNS, and cognitive or behavioral deficits [21–23]. However, because of its parallels with HIV pathogenesis, the traditional SIV macaque model is hindered by the low rate of development of SIV encephalitis (SIVE) and the long time-period for its evolution. Only approximately 25% of infected macaques develop encephalitis and progression to terminal AIDS may take several years [21, 24]. These factors make it difficult for use in testing specific hypotheses.

Therefore, attention has focused on rapid progressing SIV macaque models. The accelerated model used in our study retains the use of the SIV-infected rhesus macaque, but uses a monoclonal antibody to deplete the animal of CD8+ lymphocytes [25, 26]. In this model, 80% of persistently CD8-depleted animals develop SIVE, with a course of progression to terminal AIDS within 12 weeks [27]. Recent modifications in applying the antibody has resulted in >90% of macaques becoming persistently CD8-depleted with nearly all of them developing SIVE. This model also produces profound neuronal injury detectable within weeks by in vivo ^1^H MRS [18]. The accelerated SIV macaque model combined with magnetic resonance spectroscopy (MRS) has proven to be highly informative in characterizing SIV progression from viral infection to neuronal injury and offers valuable insight into HAND’s therapeutic conundrum [28].

Using *in vivo* MRS, the neuronal marker N-acetylaspartate/creatine (NAA/Cr) enables temporal monitoring of neuronal integrity as disease progresses. Upon SIV infection, NAA/Cr levels steadily decline [17, 18].Various studies have shown lower NAA/Cr levels in neurocognitively-impaired HIV+ individuals compared to neuroasymptomatic subjects, specifically in the basal ganglia and frontal cortex [29–31]. The extent of NAA decline in those affected by HAND has been associated with severity of neurocognitive impairment [32]. The SIV model also allows for *postmortem* confirmation of compromised neuronal health by immunohistochemical staining for microtubule-associated protein 2 (MAP2) and synaptophysin (SYN), markers for synaptodendritic integrity. In human studies, the extent of damage to the synaptodendritic structures has been shown to correlate strongly with level of neurocognitive impairment [16, 33–36].

The purpose of our investigation was to quantitatively analyze the relationship between the amount of virus in the brain, CSF and plasma and the severity of the resultant neuronal injury. Towards that, 23 animals were infected with SIVmac251 virus and depleted of CD8^+^ T lymphocytes using anti-CD8 antibody to accelerate progression to neuroAIDS. Disease progression was modulated either with cART (4 animals) or minocycline (7 animals). cART consisted of 9-R-2-Phosphonomethoxypropyl adenine (PMPA), 5-Fluoro-1-[(2R,5S)-2-(hydroxymethyl)-[1,3]oxathiolan-5-yl]cytosine (FTC), and 2'-3'-didehydro-2'-3'-dideoxythymidine (Stavudin). The rationale of using this particular cART regimen was based on a study by zur Megede et al. [37] which resulted in a significant drop in viral loads in their SIVmac239/macaque model. Minocycline (MN), a well-tolerated and inexpensive antibiotic that has been tested on a variety of neurodegenerative diseases including HAND, has been found to have anti-inflammatory/neuroprotective effects and, possibly, a direct anti-HIV effect [38–40]. Previously, our group has shown that minocycline reduces trafficking of infected monocytes into the brain [41].

Our group found a direct relationship between the amount of brain viral load assessed using reverse transcription Polymerase chain reaction (RT-PCR) and the severity of neuronal injury assess by *in vivo* magnetic response spectroscopy. Furthermore, reduction of brain virus load by combination anti-retroviral therapy and by minocycline produced neuroprotection in this primate model of AIDS.

## Materials and methods

### Experimental design

Twenty-seven rhesus macaques were included in this study. Four animals served as controls for the *postmortem* evaluations. Twenty-three animals were infected with SIVmac251 virus and depleted of CD8^+^ T lymphocytes using anti-CD8 antibody. 12 animals remained untreated and were sacrificed at 4, 6 and 8 weeks post inoculation (wpi). Eleven animals were treated with either cART (4 animals) starting at 6 wpi or minocycline (7 animals) starting at 4 wpi. MRI and MRS was performed on 23 animals twice pre-inoculation and biweekly until sacrifice. **S1 Fig** illustrated the experimental design. Activated CD14+CD16+ monocytes quantified by flow cytometry, viral RNA in the plasma and cerebrospinal fluid (CSF) was quantified by RT-PCR. *Postmortem*, neuronal integrity was evaluated by quantitative IHC using MAP2 and SYN, and viral RNA in the brain (frontal cortex) was quantified by RT-PCR.

### Ethics statement

This study was carried out in strict accordance with the recommendations in the Guide for the Care and Use of Laboratory Animals of the National Institutes of Health. The Massachusetts General Hospital (MGH) Subcommittee on Research Animal Care (SRAC) and the Institutional Animal Care and Use Committee (IACUC) of Harvard University reviewed and approved the animal care and protocol. Specifically, the MGH SRAC approved protocol number 2003N000167 and the Harvard University IACUC approved protocol number 3076. All animal studies were performed in accordance with federal laws and regulations, international accreditation standards, and institutional policies.

Animals were housed according to the standards of the American Association for Accreditation of Laboratory Animal Care. Treatment of animals was in accordance with the Guide for the Care and Use of Laboratory Animals of the Institute of Laboratory Animal Resources. Animals were quarantined at the New England Primate Research Center (NEPRC) and transported to the Center for Comparative Medicine (CCM) for the study. Housing in a laboratory area was inspected and approved by both the SRAC and the Director of CCM.

All animals received environmental enrichment and were monitored daily for evidence of disease and changes in attitude, appetite, or behavior suggestive of illness. Specifically, animals were clinically monitored for general health with complete blood counts and physical examinations performed. Weekly observations of body weight, food consumption and stool character was also recorded. Health checks were performed each morning on every animal at the MGH CCM by the animal care staff and if a problem was noted the animal was examined by a veterinarian. The monkeys were on a diet of high-protein monkey diet biscuits.

Appropriate clinical support was administered under the direction of the attending veterinarian and included analgesics, antibiotics, intravenous fluids, and other supportive care. Animals were euthanized when they presented with advanced stages of AIDS; criteria for euthanasia included 15% weight loss in two weeks, unresponsive opportunistic infection, persistent anorexia, intractable diarrhea, progressive neurologic signs, significant cardiac or pulmonary signs or other serious illness.

Animals were euthanatized at the NEPRC at the end of the study, specifically within 24 hours after their final MRI/MRS studies. Euthanasia was performed by administering a lethal dosage of pentobarbital sodium (100 mg/kg, IV). For the purposes of this study, the entire brain was examined *postmortem* quantitative histopathology, rendering death as an endpoint.

### Non-human primates

Twenty-seven (27) Indian-origin adult (4–5 year-old) rhesus macaques (*Macaca mulatta*) were included in this study. All macaques were tested to be specific pathogen free (SPF-free). Twenty-three (23) animals were infected with SIVmac251 virus (20 ng SIVp27, i.v.) and depleted of CD8^+^ T lymphocytes using anti-CD8 antibody cM-T807 at 6, 8 and 12 days post inoculation (dpi) [18, 25, 26]. Four animals served as controls for the *postmortem* evaluations and underwent CD8^+^ T cell depletion. Of note, all animals were age-matched and infected with the same SIVmac251 virus batch.

Twenty-three animals were scanned twice pre-inoculation and biweekly until sacrifice. During MR scanning sessions, each animal was tranquilized with 15–20 mg/kg intramuscular ketamine hydrochloride and intubated to ensure a patent airway during the experiment. Intravenous injection of 0.4 mg/kg atropine was administered to prevent bradycardia, and continuous infusion of approximately 0.25 mg/kg/min propofol was maintained throughout the experiment via catheter in a saphenous vein. Heart rate, oxygen saturation, end-tidal CO_2_ and respiratory rate were monitored continuously. A heated water blanket was used to prevent hypothermia.

Four animals were treated with cART consisting of 9-R-2-Phosphonomethoxypropyl adenine (PMPA), 5-Fluoro-1-[(2R,5S)-2-(hydroxymethyl)-[1,3]oxathiolan-5-yl]cytosine (FTC), and 2'-3'-didehydro-2'-3'-dideoxythymidine (Stavudine, Zerit®) starting at 42 dpi. PMPA and FTC were administered subcutaneously at daily dosages of 30 mg/kg and 50 mg/kg, respectively. PMPA and FTC were provided by Gilead Sciences, Inc. (Foster City, CA.) via a material transfer agreement. Zerit was administered orally at 1.2 mg/kg twice daily [37]. Seven animals were administered minocycline orally starting at 28 dpi at daily dosage of 4 mg/kg b.i.d. [40].

Cohorts are differentiated on basis of 1) time period between SIV-inoculation and sacrifice, 2) treatment versus none and 3) length of CD8-depletion (persistent versus short-term) (see **Table 1**). The three untreated cohorts include four animals sacrificed at 4 wpi, four animals sacrificed at 6 wpi and four animals sacrificed at 8 wpi. Treated cohorts include four animals treated with cART from 6–12 wpi and seven minocycline-treated animals. Minocycline-treated animals are further divided into those persistently CD8-depleted (n = 4) versus short-term CD8-depleted animals (n = 3). All other cohorts consisted of persistently CD8-depleted animals, defined as CD8 lymphocyte depletion for greater than 28 dpi.

**Table 1:** Cohorts of SIV-infected animals.

N	CD8 depletion	Sacrificed	Treatment	Treatment initiation
**4**	persistent	4 wpi*	N/A	-
**4**	persistent	6 wpi	N/A	-
**4**	persistent	8 wpi	N/A	-
**4**	persistent	12 wpi	cART^#^	6 wpi
**4**	persistent	8 wpi	Minocycline	4 wpi
**3**	short-term	8 wpi	Minocycline	4 wpi

*WPI: week post-inoculation

^#^ cART: PMPA, FTC and Stavudine.

### Magnetic resonance spectroscopy

MRI/MRS experiments were performed on a 3 Tesla whole-body imager (Magnetom TIM Trio, Siemens AG, Erlangen, Germany), using a circularly polarized transmit-receive extremity coil. A three-plane localizer scan was used to position the monkey in the coil, ensuring highly reproducible voxel placement. To guide placement of the ^1^H-MRS volumes of interest, sagittal and axial turbo spin echo (TSE) images were obtained using the following parameters: 140×140 mm^2^ field of view, 512×512 matrix, TE (echo time) of 16 ms; slice thickness was 2 mm for sagittal images and 1.2 mm for axial images; TR (repetition time) was 4500 ms for sagittal and 7430 ms for axial images resulting in an acquisition time of 3 minutes for the sagittal and 5 minutes for the axial TSE sequence.

Single voxel ^1^H MR spectroscopy was performed in the frontal cortex (FC) among other regions using a point-resolved spectroscopy sequence (PRESS) with water suppression enhanced through T1 effects (WET), and the following parameters: TE = 30 ms, TR = 2500 ms, and 192 acquisitions resulting in an acquisition time of 8 minutes. All spectra were processed offline using the LCModel software package [42] to determine the quantities of the brain metabolites NAA and Cr. Absolute metabolite concentrations in institutional units were derived from the same voxel using the water signal as reference. In this study, our results focus on the frontal cortex as we have seen the most prominent metabolic changes in the cortex in the SIV-infected macaque model.

### Viral load analysis

Plasma and cerebrospinal fluid SIV RNA was quantified using real-time reverse transcription-PCR [43]. Blood was centrifuged at 20,000 g for 1 hour, and CSF samples were stored at -80°C until analysis. The threshold sensitivity was 100 copy eq./mL. Results are averages of duplicate determinations.

Brain tissue RNA was isolated using an RNeasy MiniKit from Qiagen (Valencia, CA). 50 mg of brain tissue from the frontal cortex of each animal was used. The protocol provided by the manufacturer was followed. Purified RNA was analyzed by RT-PCR.

### Flow cytometry

Peripheral blood was drawn twice prior to infection, at days 6, 8, and 12 pi, and weekly thereafter. Flow cytometric analyses were performed with 100 μl samples of blood as previously described [41]. Fluorochrome-conjugated primary antibodies including anti-CD3-FITC (SP34-2), anti-CD4-FITC (L200), anti-CD8-PE (DK25; Dako), anti-CD14-FITC (M5E2), anti-CD16-PE (3G8), all from BD Pharmingen were used. Samples were fixed in PBS containing 2% formaldehyde, acquired on a FACSAria cell sorter (Becton-Dickinson) and analyzed with Tree Star Flow Jo version 8.7. Monocytes are first selected based on size and granularity (FSC vs. SSC). From this gate, HLA-DR^+^ CD14^+^ monocytes were selected. Complete blood counts were obtained using a CBC Hematology Analyzer (Hema-True, HESKA). The absolute number of CD8^+^ lymphocytes was determined by multiplying the percentage of CD8^+^/CD3^+^ cells by absolute lymphocyte counts obtained using a standard veterinary 3-point WBC differential, CBC Hematology Analyzer. The absolute number of peripheral blood monocytes was calculated by multiplying the total white blood cell count by the total percentage of each monocyte subset population as determined by flow cytometric analysis.

### Tissue collection and processing

At study endpoints animals were anesthetized with ketamine-HCl and sacrificed by intravenous pentobarbital overdose. At necropsy, animals were exsanguinated and were perfused with 4 L of chilled saline. A complete set of CNS and peripheral tissues were collected in 10% neutral buffered formalin, embedded in paraffin and sectioned at 6 μm.

### Quantitative immunohistochemistry

To evaluate synaptodendritic integrity of neurons, sections from frontal cortex were immunolabeled overnight with monoclonal antibodies, followed by biotinylated horse anti-mouse immunoglobulin G and avidin-horseradish peroxidase (Vectastain Elite kit; Vector, Burlingame, CA), then reacted with 3,3’-Diaminobenzidine (DAB). SYN is a 38-kd calcium-binding protein localized to synaptic vesicles and is commonly used to assess synaptic density [44]. MAP2 is a high-molecular-weight protein that localizes to the dendritic compartment of neurons and is involved in microtubule assembly. Monoclonal antibodies against SYN and MAP2 were used to evaluate synaptic (1:10) and dendritic integrity, respectively (Boehringer Mannheim, Indianapolis, IN). Expression of SYN and MAP2 were determined by computer image analysis, as previously described [45]. Immunoreactivity was semi-quantitatively assessed as corrected optical density by using a microdensitometer (Quantimet 570C; Leica, Microsystem Cambridge, UK). Three immunolabeled sections were analyzed from each animal. As previously described [45–47], the system was first calibrated with a set of filters of various densities and ten images for each section at 100x magnification were obtained. After delineating the area of interest (layers 2–5) with the cursor, the optical density within that area was obtained. The optical density in each image was then averaged and expressed as the mean per case. All measurements for SYN and MAP2 are in arbitrary optical density units and range from 0 to 500 (i.e. 0 indicates all light is allowed to pass through the sample, while 500 indicates no light is allowed to pass through the sample). All values are expressed as mean ± standard error of the mean.

### Statistical methods

All analyses were conducted using JMP 12.0 (SAS, Cary, NC). To determine significant differences between cohorts’ mean values at endpoints analysis of variance (ANOVA) was used; if significant by ANOVA (P<0.05) least square means Student’s t-tests were used to isolate difference between groups. For the serial data such as *in vivo* MRS and plasma or CSF viral loads, repeated measures analysis of variance (RM-ANOVA) in combination with Holm's t-tests was employed to isolate differences between time-points within the cohorts. Correlations using endpoint data were determined using Spearman Rank analysis (R_ρ_). To analyze relationships among *in vivo* markers using serial data, a least-squared means model was used. A p value of <0.05 was considered significant.

## Results

### Animal cohorts

Twenty-seven rhesus macaques were studied (**Table 1**). Twenty-three of these animals were SIV infected and underwent CD8^+^ T cell depletion [18]. Twenty animals were persistently CD8^+^ lymphocyte-depleted for a minimum of 28 days; however, three animals were short-term depleted unexpectedly, *i*.*e*. their CD8^+^ lymphocytes returned to baseline by 28 days. Twelve of the 23 animals remained untreated and sacrificed at 4, 6, and 8 weeks post inoculation (4 animals in each cohort). Four animals were treated with cART, and seven animals were treated with minocycline (MN). The cART regimen was based on a study by zur Megede et al. [37] and consisted of 9-R-2-Phosphonomethoxypropyl adenine (PMPA), 5-Fluoro-1-[(2R,5S)-2-(hydroxymethyl)-[1,3]oxathiolan-5-yl]cytosine (FTC), and 2'-3'-didehydro-2'-3'-dideoxythymidine (Stavudine, Zerit®) administered daily starting at 6 weeks post inoculation (wpi) for 6 weeks. Minocycline was administered daily starting at 4 wpi for 4 weeks. Each of the 23 infected animals were evaluated at baseline and biweekly following infection using 3T MRI neuroimaging as well as studies of blood and CSF. Four uninfected animals served as controls and did undergo CD8^+^ T cell depletion as previously described. Additionally, *postmortem* evaluation of brain tissue for all 27 animals was performed at the end of the study.

### Untreated animals

#### Viral RNA and CD14^+^/CD16^+^ monocytes

**Fig 1:**
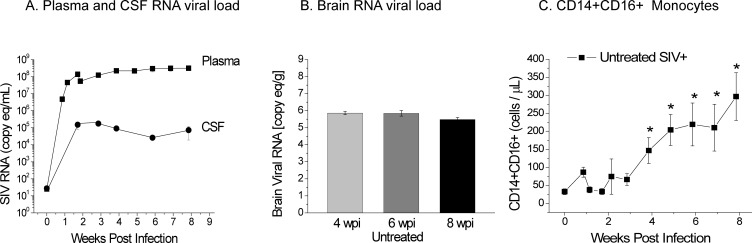
Viral RNA plateaus w/o treatment. A. Viral RNA was detectable in plasma 6 days after infection (~10^7^ copies eq./mL) and the viral load approaches a plateau by 4 weeks after infection. The mean plasma viral load was 4.9 x 10^8^ copies eq./mL at 8 wpi. Cerebrospinal fluid viral loads were found to be approximately 3 orders of magnitude lower than plasma. B. The amount of viral RNA in the frontal cortex of untreated animals was 7.5 x 10^5^, 9.2 x 10^5^ and 3.0 x 10^5^ copies eq./g at 4, 6, and 8 weeks after infection, respectively, and was not significant (P > 0.11) and had a mean of ~ 10^6^ copies eq./g. C. SIV+ animals had high levels of circulating CD14^+^/CD16^+^ monocytes 28 days after infection, and they had ~300 cells/μL by 8 weeks. (* indicates significant differences from pre-infection).

In SIV infected, CD8 depleted (SIV^+^/CD8^-^)animals, viral RNA was detectable in plasma 6 days after infection (~10^7^ copies eq./mL), and the viral load approached a plateau by ~2 wpi. The mean plasma viral load was 4.9 x 10^8^ copies eq./mL at 8 wpi (**Fig 1A**). CSF viral loads were found to be approximately three logs lower than plasma viral loads in untreated animals (**Fig 1A**). The CSF viral load in untreated animals at 8 wpi (endpoint) was 7.2 x 10^4^ copies eq./mL.

The amount of viral RNA in the frontal cortex of untreated animals sacrificed at 4, 6 and 8 wpi was 7.5 x 10^5^ copies eq./g, 9.2 x 10^5^ copies eq./g, and 3.0 x 10^5^ copies eq./g, respectively (**Fig 1B**). Viral RNA of untreated SIV^+^/CD8^-^ animals was found to be not significantly different at the various time points (P > 0.11).

SIV progression in the accelerated model is characterized by the expansion of activated CD14^+^/CD16^+^ monocytes outside the CNS, and there is much evidence that these cells play a major role in the trafficking of virus across the BBB into the brain [18, 41]. SIV^+^ animals had high levels of circulating CD14^+^/CD16^+^ monocytes 28 days after infection and ~300 cells/μL by 8 wpi (**Fig 1C**).

#### Neuroimaging and neuropathology

**Fig 2:**
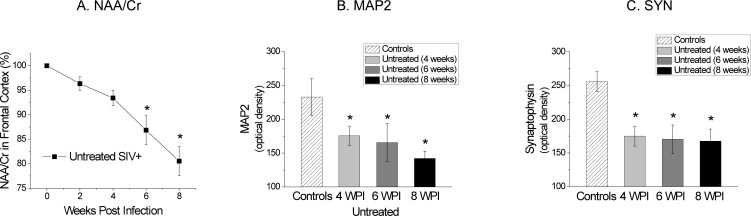
MRS and IHC show neuronal injury in infected animals w/o treatment. A. The neuronal marker N-Acetylasparate/creatine steadily declined following SIV infection in all animals, reaching decreased as low as 20% below baseline by 8 wpi in untreated animals. (* indicates significant changes from baseline levels prior infection.) B. Average microtubule-associated protein 2 levels in the frontal cortex of SIV+ untreated animals sacrificed at 4 wpi, 6 wpi and 8 wpi were lower compared to uninfected control animals. (* indicates significant differences from controls.) C. Average synaptophysin levels in the frontal cortex of SIV+ untreated animals sacrificed at 4 wpi, 6 wpi and 8 wpi were lower compared to uninfected control animals. (* indicates significant differences from controls).

Brain MRI was performed biweekly and no structural or signal abnormality was identified in any animal; however, major metabolic changes were observed by ^1^H MR spectroscopy. The neuronal marker NAA/Cr steadily declined following SIV infection in all animals, reaching decreases as low as 20% below baseline by 8 wpi in untreated animals **(Fig 2A**). The decrease in NAA/Cr following infection is due to both, decreases in neuronal NAA and increases in Cr, which most likely reflects the cumulative effects of altered metabolic states of neurons and glial cells, respectively, in the setting of SIV infection (for further discussion, see Ratai et al. [17].

To confirm the relationship between *in vivo* MRS findings and neuronal injury, we performed *postmortem* quantitative immunohistochemical measurements of SYN, a marker of pre-synaptic neuronal damage, and MAP2, a marker of post-synaptic neuronal damage. Mean MAP2 levels in the frontal cortex of SIV+ untreated animals were 176 at 4 wpi, 166 at 6 wpi and 142 at 8 wpi (**Fig 2B**). MAP2 levels of SIV+ untreated animals sacrificed at 4, 6 and 8 wpi were decreased compared to uninfected controls (P = 0.05, P = 0.02, and P = 0.004, respectively). While MAP2 expression decreased with disease progression, there was no significant difference between in MAP2 4, 6 and 8 wpi.

Mean levels of SYN in the frontal cortex of SIV+ untreated animals are shown in **Fig 2C**. One animal was excluded from the SYN analysis as it was identified as an outlier by Grubb’s Test using JMP 12.0. Mean SYN levels were 175 at 4 wpi, 170 at 6 wpi and 168 at 8 wpi. Decreased levels of SYN were observed in untreated animals sacrificed at 4, 6, and 8 wpi controls (P = 0.0058, P = 0.0067, and P = 0.0030, respectively) compared to uninfected controls. There was no significant difference in SYN between 4, 6 and 8 wpi in SIV-infected macaques.

### CART treated animals

#### Viral RNA and CD14^+^/CD16^+^ monocytes

In a prior study using PMPA, FTC, and stavudine, all infected animals remained below 10^4^ RNA copies/ml after cART treatment [37]. Thus, we used a similar combined antiretroviral treatment for SIV^+^/CD8^-^ animals, however treated much shorter. The rationale for administering cART for only 6 weeks was based on our previous studies in which SIV infected/CD8 depleted animals received cART (consisting of the nonpenetrating agents PMPA and RCV) for four weeks, beginning 4 weeks after SIV inoculation (43) (18). Typically, studies using CD8-depleted, SIV-infected macaques are brief in duration due to the rapidly progressive course of SIV infection in these experimentally immunocompromised animals, and this had to be taken into consideration when the experiment was designed.

**Fig 3:**
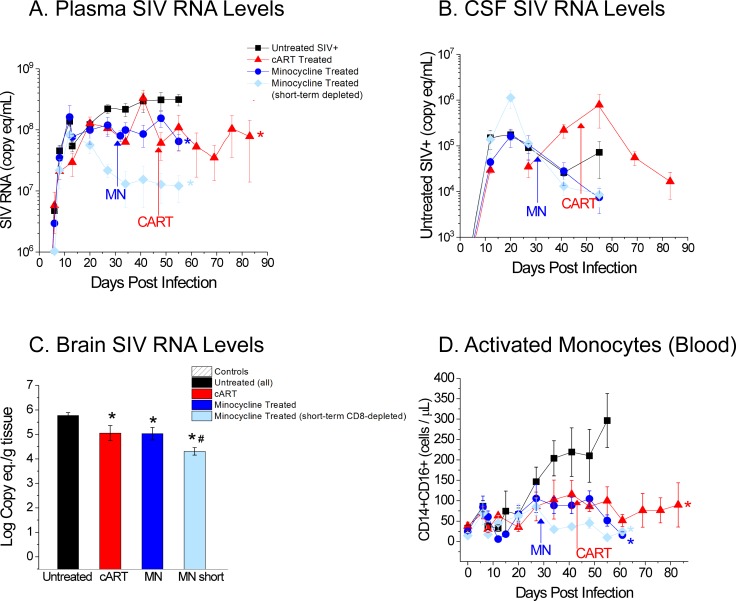
Viral levels in the three compartments and activated monocytes with and w/o treatment. A. Treated animals revealed lower plasma vRNA levels (~1 log) at endpoints when compared to untreated animals at 8 wpi (cART P = 0.019; MN_persistent_ P = 0.0063). Lowest plasma SIV RNA levels (~2 log compared to untreated) were observed in animals that were minocycline treated and had partial immune reconstitution of the CD8^+^ T cell population (P = 0.0032). (* indicates statistically significant differences when compared to untreated animals.) B. Cerebrospinal fluid viral levels decreased in cART and in minocycline treated animals after endpoint, yet differences in cerebrospinal fluid viral burden failed to reach significance. C. Viral levels in cART and minocycline treated animals were lower than those in untreated animals (cART P = 0.007; MN_persistent_ P = 0.006; MN_short_ P< 0.0001). Short-term CD8-depleted minocycline-treated animals had significantly reduced brain vRNA levels when compared to all other cohorts, specifically in animals that were minocycline treated and CD8-depleted (P = 0.032). (* indicates statistically significant differences when compared to untreated animals. # indicates statistically significant differences when compared to all other cohorts.) D. The cART cohort and the minocycline treated cohort had significantly lower CD14^+^/CD16^+^ monocytes compared to untreated at their endpoints (cART P = 0.014; MN_persistent_ P = 0.004; MN_short_ P = 0.0035). (* indicates statistically significant differences when compared to untreated animal.).

In our cART cohort, plasma viral loads were roughly one log lower at their endpoints when compared to untreated animals at 8 wpi (7.8 x 10^7^ copies eq./mL P = 0.019, **Fig 3A**). In the cART cohort, the plasma viral load also appears to stabilize after 6 weeks of cART. As compared to zur Megede’s study, modest reduction was seen in the plasma viral load due to short-term use of cART and CD8 depletion [37]. CSF vRNA was decreased in the cART cohort (1.7 x 10^4^ copies eq./mL) at endpoint compared to untreated animals at 8 wpi (7.2 x 10^4^ copies eq./mL, **Fig 3B**); however, differences in CSF viral burden did not reach statistical significance.

Brain tissue vRNA levels in short-term cART treated animals were lower than those in untreated animals (1.1 x 10^5^ copies eq./g, P = 0.007, **Fig 3C**). These findings are consistent with our previous reports on PMPA- and RCV-treated animals [18, 48].

Treatment with cART also prevented an increase of circulating activated CD14^+^/CD16^+^ monocytes. The cART cohort had significantly lower CD14^+^/CD16^+^ monocytes compared to the untreated cohort at endpoints (P = 0.014, **Fig 3D**).

#### Neuroimaging and neuropathology

**Fig 4:**
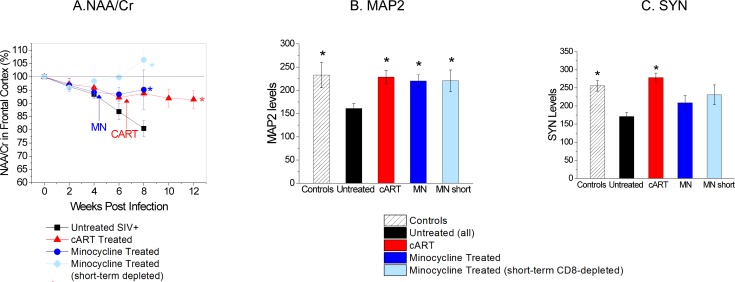
Viral RNA and neuronal markers (NAA/Cr, MAP2, and SYN) with and w/o treatments. A. The decrease in NAA/Cr was arrested with this regimen of combination antiretroviral treatment as well as minocycline treatment resulting in higher NAA/Cr levels when compared to untreated animals sacrificed at 8 wpi, (cART P = 0.09; MN_persistent_ P = 0.029; MN_short_ P = 0.0012). Animals that were minocycline treated and had partial immune reconstitution of the CD8 T cell population showed the best results. B. MAP2 levels in combination antiretroviral treated and in minocycline treated animals were significantly higher than untreated SIV-infected animals (cART P = 0.006; MN_persistent_ P = 0.010; MN_short_ P = 0.016). C. Similarly, SYN levels in combination antiretroviral treated animals were significantly higher than untreated SIV-infected animals (P = 0.002). (* indicates statistically significant differences when compared to untreated animal.).

The decline in NAA/Cr was arrested with the cART treatment (**Fig 4A**) resulting in higher NAA/Cr levels when compared to untreated animals sacrificed at 8 wpi, (P = 0.09). MAP2 and SYN levels in the cART-treated cohort were significantly higher than in untreated SIV^+^ animals (P = 0.006 and P = 0.002, respectively), comparable to the four uninfected animals (**Fig 4B**).

### Minocycline treated animals

#### Viral RNA and CD14+/CD16+ monocytes

Minocycline is an anti-inflammatory treatment that has been reported to have an antiretroviral effect [38]. It has been previously noted that MN treatment decreases plasma SIV viral load [41]. In the persistently CD8-depleted MN-treated cohort, plasma viral loads were roughly one log lower at their endpoints when compared to untreated animals at 8 wpi (MN_persistent_ 6.5 x 10^7^ copies eq./mL P = 0.0063, **Fig 3A**). The plasma viral load also appears to plateau as therapy is begun. In the animals who had early CD8+ T cell recovery and who were treated with MN, there was a decline from peak viral load levels to a new plateau that was nearly two orders of magnitude lower than that of untreated animals (MN_short_ 1.2 x 10^7^ copies eq./mL P = 0.0032, **Fig 3A**). CSF vRNA was decreased in MN treated animals (MN_persistent_: 7.5 x 10^3^ copies eq./mL and MN_short_: 8.6 x 10^3^ copies eq./mL) at endpoint compared to untreated animals at 8 wpi (7.2 x 10^4^ copies eq./mL); however, differences in CSF viral burden between the MN-treated cohort and untreated animals did not reach statistical significance (**Fig 3B**).

Brain vRNA levels in MN-treated animals were lower than those in untreated animals (MN_persistent_ 1.1 x 10^5^ copies eq./g P = 0.006; MN_short_ 2.0 x 10^4^ copies eq./g P<0.0001; **Fig 3C**). Short-term CD8-depleted MN-treated animals had significantly reduced brain vRNA levels when compared to all other cohorts, including persistently CD8-depleted MN-treated animals (P = 0.032).

Treatment with MN prevented an increase of circulating activated CD14^+^/CD16^+^ monocytes. Both MN cohorts had significantly lower CD14^+^/CD16^+^ monocytes compared to untreated animals at their endpoints (MN_persistent_ P = 0.004; MN_short_ P = 0.0035). Data on MN-treated animals have previously been reported in Campbell et al. [41].

#### Neuroimaging and neuropathology

The decline in NAA/Cr was arrested with MN treatment (**Fig 4A**) resulting in higher NAA/Cr levels when compared to untreated animals sacrificed at 8 wpi, (MN_persistent_ P = 0.029; MN_short_ P = 0.0012). Animals that were MN-treated and had partial immune reconstitution of the CD8+ T cell population had the most complete recovery (**Fig 4A**). Data on the neuroprotective nature of MN on neuronal injury have been previously reported in Ratai et al. [40].

MAP2 levels in all treated cohorts were significantly higher than in untreated SIV-infected animals (MN_persistent_ P = 0.010; MN_short_ P = 0.016) and were comparable to the four uninfected animals (**Fig 4B**). There was no significant difference between the SYN levels of MN-treated and untreated SIV^+^ animals (**Fig 4C**).

### Endpoint correlations between viral loads and compartments

There was a strong correlation between the amount of viral RNA in the plasma and in the brain (P = 0.00030, R_ρ_ = 0.68). In addition, there is a significant correlation between viral loads in brain and CSF (P = 0.0080, R_ρ_ = 0.54). There was no significant correlation, however, between viral RNA in plasma and in CSF (P = 0.16; **Table 2**).

**Table 2:** Correlation between viral loads in different compartments and CD14+/CD16+ monocytes.

Endpoint Measure	Endpoint Measure	N	Spearman Rank Correlation Coefficient rho	P Value
Plasma Viral Load	Brain Viral Load	23	0.68	0.00030
CSF Viral Load	Brain Viral Load	23	0.54	0.0080
Plasma Viral Load	CSF Viral Load	23	0.30	0.16
Plasma Viral Load	CD14+/CD16+ Monocytes	23	0.61	0.018
Brain Viral Load	CD14+/CD16+ Monocytes	23	0.52	0.018
CSF Viral Load	CD14+/CD16+ Monocytes	23	0.26	0.27

Expansion of the circulating CD14^+^/CD16^+^ monocyte subset strongly correlated with viral levels in plasma (P = 0.0018, R_ρ_ = 0.61) and brain (P = 0.00030, R_ρ_ = 0.68), consistent with previous findings [18, 41, 49], but did not correlate with viral RNA in the CSF (P = 0.27, R_ρ_ = 0.26).

### Endpoint correlations between viral /loads and neuronal biomarkers

Understanding the relationship between the severity of neuronal injury and the amount of virus in all three compartments may explain the viral entry into the CNS and the subsequent fate of the virus. In our study, the severity of neuronal injury was reflected by declines of neuronal biomarkers which correlated with the amount of virus in the brain and plasma. The severity of neuronal injury also correlated with the number of activated CD14^+^/CD16^+^ monocytes, but not with the CSF viral load (**Table 3**). At endpoints, significant inverse correlations were found between all three neuronal biomarkers (NAA/Cr, MAP2, SYN) and viral levels in both brain and plasma **(S2 Fig**), indicating more severe neuronal injury with higher viral levels in plasma and brain compartments. In addition, endpoint levels of CD14^+^/CD16^+^ monocytes significantly inversely correlated with NAA/Cr and MAP2 levels. Inverse correlation between endpoint CD14^+^/CD16^+^ monocyte levels and SYN levels trended toward statistical significance. CSF viral loads had no significant correlation with any of the neuronal markers at endpoint.

**Table 3:** Correlations between viral load and neuronal markers.

**Endpoint Measure**	**Neuronal Marker**	**N**	**Spearman Rank Correlation Coefficient rho**	**P Value**
Brain Viral Load	ΔNAA/Cr	23	-0.48	0.019
Brain Viral Load	MAP2	23	-0.52	0.012
Brain Viral Load	SYN	23	-0.42	0.048
Plasma Viral Load	ΔNAA/Cr	23	-0.48	0.019
Plasma Viral Load	MAP2	23	-0.47	0.022
Plasma Viral Load	SYN	23	-0.47	0.024
CD16+/14+ Blood Monocytes	ΔNAA/Cr	23	-0.44	0.035
CD16+/14+ Blood Monocytes	MAP2	23	-0.44	0.034
CD16+/14+ Blood Monocytes	SYN	23	-0.35	0.10
CSF Viral Load	ΔNAA/Cr	23	-0.19	0.39
CSF Viral Load	MAP2	23	-0.25	0.26
CSF Viral Load	SYN	23	-0.23	0.30
**Longitudinal Measure**	**Neuronal Marker**	**N**	**Correlation Coefficient R**	**P Value**
Plasma Viral Load	ΔNAA/Cr	88	-0.41	0.0053
CD14+CD16+ Blood Monocytes	ΔNAA/Cr	85	-0.49	0.0001
CSF Viral Load	ΔNAA/Cr	88	-0.30	0.17

### Longitudinal correlations between viral loads and neuronal biomarkers

In the longitudinal data analysis of the study, neuronal biomarker NAA/Cr shows a strong inverse correlation with plasma viral load (P = 0.0053, R_ρ_ = -0.41) and levels of activated CD14^+^/CD16^+^ monocytes (P = 0.0001, R_ρ_ = -0.49). No correlation was found between NAA/Cr and CSF viral load (P = 0.17, R_ρ_ = 0.30; **Table 3**).

## Discussion

The accelerated (*i*.*e*., CD8 lymphocyte-depleted) SIV-infected macaque model recapitulates key features of HIV neuropathogenesis [18, 25, 26]. Immunodeficiency virus-induced neuronal injury is highly reproducible using this model, thus allowing scrutiny of the complete chain of events underlying HAND progression in the same living animal. During SIV progression, viral loads were measured in three compartments (e.g. plasma, CSF, and brain) and neuronal health was assessed longitudinally by the *in vivo* MRS marker NAA/Cr. Neuronal structural integrity was assessed *postmortem* by immunohistochemical staining for SYN and MAP2, markers of synaptodendritic integrity. SIV progression was manipulated by the following three treatment conditions: 1) cART (PMPA, FTC, and stavudine), 2) minocycline, 3) minocycline in the setting of incomplete immune suppression.

### Viral loads plateaued early after infection and CD8 depletion

In this primate model, plasma viral load reached high levels and then plateaued. The amount of virus in the brain was approximately two logs lower than in plasma and did not change significantly after blood levels plateaued at 4 wpi. Finally, the lowest viral loads were observed in the CSF. Therefore, the relative vRNA concentrations within each compartment and the correlations between viral loads (shown in **Table 2**) suggest a trajectory of the virus from blood to brain to CSF. Furthermore, the observed plateauing of plasma, brain, and CSF viral loads suggests a balance in production of virus in blood was attained. Thus, we hypothesize that the production of virus within each compartment is balanced by clearance mechanisms resulting in the observed “steady-state” levels. One clearance mechanism may constitute the movement of virus from one compartment to the other. In the cART and MN treatment cohorts, the plasma viral load also appeared to plateau as therapy began, which suggests that a different “steady state” was attained with a new balance between viral production and removal.

Lack of correlation between plasma and CSF viral loads in our SIV model supports observations of discordant levels of HIV in plasma and CSF both in neuroasymptomatic and neurocognitively-impaired individuals receiving antiretroviral treatment [50, 51]. Our study comparing viral levels in three different compartments raises the possibility of preferential influx of virus from plasma to CNS parenchyma and subsequent harboring of the virus with potential for persistent seeding to the CSF and/or plasma.

### Neuronal injury strongly correlated with CNS vRNA

SIV-induced alterations in neuronal viability, as assessed *in vivo* and confirmed *postmortem*, were found to correlate with the amount of vRNA in the corresponding region. In SIV-infected animals that were not treated, *in vivo* brain neuronal marker levels continued to decrease despite a plateau in viral loads in the three compartments. Thus, neuronal injury appears to be cumulative.

The provision of MN or cART, however, stabilized markers for neuronal viability indicating there is no further damage to neurons or, alternatively, that the rate of neuronal injury caused by the continuing CNS infection is balanced by endogenous neuronal recovery mechanisms. *Postmortem* studies using immunohistochemical staining for SYN and MAP2 revealed that SIV-induced alterations are reversible after the initiation of cART and MN treatment. These results are consistent with our prior studies [18, 40].

### Peripheral factors such as plasma vRNA levels and activated monocytes are critical factors in CNS infection and injury

In this accelerated SIV model, levels of vRNA in CNS parenchyma strongly correlate with plasma vRNA levels, implicating peripheral viral replication as a critical factor in CNS infection. Furthermore, unsuppressed plasma viral levels corresponded with increased production of activated monocytes in the periphery, which have been shown to play a major role in trafficking the virus into the CNS [18, 41, 52, 53] and to correlate with neurocognitive impairment in the setting of HIV [54].

### Control of peripheral factors can ameliorate CNS injury

As compared to zur Megede’s study, one interesting observation in our study was that the utilized cART did not very effectively decrease the viral loads [37]. However, despite the modest reduction in plasma viral load by cART, neuronal injury was alleviated. Both cART and MN inhibited activated monocyte expansion, and these changes paralleled recovery of NAA/Cr levels in the brain. This suggests that manipulation of peripheral factors can have a significant impact on the progression of neuronal injury.

Our findings, if projected to HIV-infected humans, may offer valuable insight into HAND’s therapeutic conundrum, discussed below:

### Relationship between CNS vRNA and neuropsychological functioning in HIV-1 infection

The observed plateauing of the brain viral load in the primate model may explain prior reports of poor correlation between CNS viral levels and neurocognitive status of HIV-infected individuals [15, 55–57]. While viral loads seem to plateau, neuronal marker levels continued to decrease, indicating that neuronal injury is cumulative.

### Effectiveness of AZT

The modest effectiveness of cART treatment helps explain the observations of neurocognitive recovery with the use of anti-retrovirals with poor CNS penetrance [58]. These include the landmark study that demonstrated HIV-associated cognitive abnormalities are partially ameliorated after the administration of zidovudine (AZT), an anti-retroviral drug with poor CNS penetrance [58]. The lowering of plasma viral load by such drugs should reduce the viral flux into the CNS with a resultant lower viral load in that compartment, allowing neuronal recovery.

### Era of highly active antiretroviral therapy (HAART)

We hypothesize that the time course and cumulative nature of neuronal injury seen in this model may also suggest a mechanism for the slowly progressive neurocognitive changes observed in the current era of highly active antiretroviral therapy [59–61]. While HAART therapy may greatly suppress viremia, imperfect adherence to therapy or other factors may lead to transient increases in viremia with a resultant increase of virus into CNS compartments. Such increases may temporarily overcome constituent neuroprotective mechanisms. While a single episode may be clinically silent, the cumulative effect over many years could produce clinically observable cognitive dysfunction.

### Failure of clinical trials using neuroprotective agents

In HIV studies, degree of neurocognitive impairment has been shown to correlate with reduced NAA levels [32]. Although neuronal apoptosis has been observed in brains of individuals with HAND [62, 63], the general consensus is that prolonged neuronal dysfunction precipitates permanent neuronal loss. Reversible alterations in synaptodendritic networks have been demonstrated in the setting of HIV infection [16, 64], and damage to these structures is a strong pathological correlate of neurocognitive impairment due to HIV [33, 65].

The time course of reversible and permanent neuronal injury may also provide an experimental design explanation for the failures of clinical trials conducted to date in which neuroprotective and other drugs were used as adjunct therapy to ameliorate neurocognitive deficits in HIV-infected individuals. Such trials typically involve treatment that last up to 20 weeks [66, 67]. It is likely that the neurocognitive dysfunction in HIV-infected individuals is due both to permanent neuronal injury that has accumulated over years and as well as some degree of recoverable injury that has occurred recently. It would be expected that a neuroprotective drug would help reverse the recoverable but not the permanent components of injury. If the permanent injuries are much greater than the recoverable ones, then failure in improving neurocognitive measurements are to be expected. A more rational design of clinical trials that account for the CNS viral load and neuronal damage may produce better outcomes.

### Interference strategies

Our study also suggests that the interference of viral trafficking into the CNS may be a useful approach, especially therapies targeting CD14^+^/CD16^+^ monocytes [18, 52–54, 57, 68]. The severity of neuronal injury strongly correlated with the expansion of CD14^+^/CD16^+^ monocytes, suggesting HAND is instigated by this peripheral monocyte subset. Expansion of the CD14^+^/CD16^+^ monocytes has been shown to correlate with lower neuropsychological performance and CNS injury in HIV+ individuals [54, 68] and with CNS disease in the setting of SIV infection [18]. Activated monocytes’ clear pathogenic role in HAND suggests that effective HAND treatments may require incorporation of monocyte-directed therapies targeting this potential peripheral viral reservoir, as proposed by Valcour et al [69].

Potential approaches include interference of virally infection monocytes, which appears to explain in part the efficacy of MN as a neuroprotective agent [38, 41]. Minocycline, which was previously shown to suppress SIV-induced encephalitis [70, 71], was also able to reduce viral loads in the brain [38]. An effective approach is to use antiretrovirals that can penetrate the blood brain barrier [6, 72].

Finally, a combination of drugs that target different points in this chain of events may be the most effective strategy. Future studies will include the use of antiretroviral therapy in combination with MN or other neuroprotective drugs.

### Summary and relevance for the future

In the accelerated SIV model, there is significant turnover of replicating virus within the brain. Additionally, the severity of neuronal injury is directly related to the brain viral load and may not be reflected by CSF viral levels. Such injury is reversible via reduction of brain viral levels. Targeting plasma viral load and disruption of trafficking of the virus into the CNS compartment via activated monocyte/macrophages are two potential approaches to preserving and/or reversing neuronal injury. Trafficking of the virus from plasma to the CNS parenchyma via activated monocytes may explain several uncertainties regarding the state of the CNS in those infected by HIV and offers valuable insight into strategies to promote CNS health.

## Supporting information

S1 Fig Study design. Twenty-three animals were infected with SIVmac251 virus and depleted of CD8+ T lymphocytes using anti-CD8 antibody. 12 animals remained untreated and were sacrificed at 4, 6 and 8 weeks post inoculation (wpi). Eleven animals were treated with either cART (4 animals) starting at 6 wpi or minocycline (7 animals) starting at 4 wpi. MRI and MRS was performed twice pre-inoculation and biweekly until sacrifice.
(PDF)

S2 Fig Correlations between viral loads and neuronal markers at endpoint. A. Plasma Viral Load was observed to be inversely correlated with percent changes in N-Acetylasparate/Creatine (R_ρ_ = -0.48, P = 0.019). B. Plasma Viral Load was inversely correlated with microtubule associated protein 2 (R_ρ_ = -0.47, P = 0.022) at endpoint. C. Plasma Viral Load was negatively correlated with synaptophysin (R_ρ_ = -0.47, P = 0.024) at endpoint. D. Brain Viral Load was shown to be inversely correlated with percent changes in N-Acetylasparate/Creatine (R_ρ_ = -0.48, P = 0.019) at endpoint. E. Brain Viral Load was observed to be negatively correlated with microtubule associated protein 2 (R_ρ_ = -0.52, 0.012) at endpoint. F. Brain Viral Load was inversely correlated with synaptophysin (R_ρ_ = -0.42, P = 0.048) at endpoint.
(PDF)
