# Supplementary figures and images for: Temporal/compartmental changes in viral RNA and neuronal injury in a primate model of NeuroAIDS
Source: PLoS One. 2018 May 11;13(5):e0196949. doi: 10.1371/journal.pone.0196949 (PMC5947913; doi:10.1371/journal.pone.0196949)

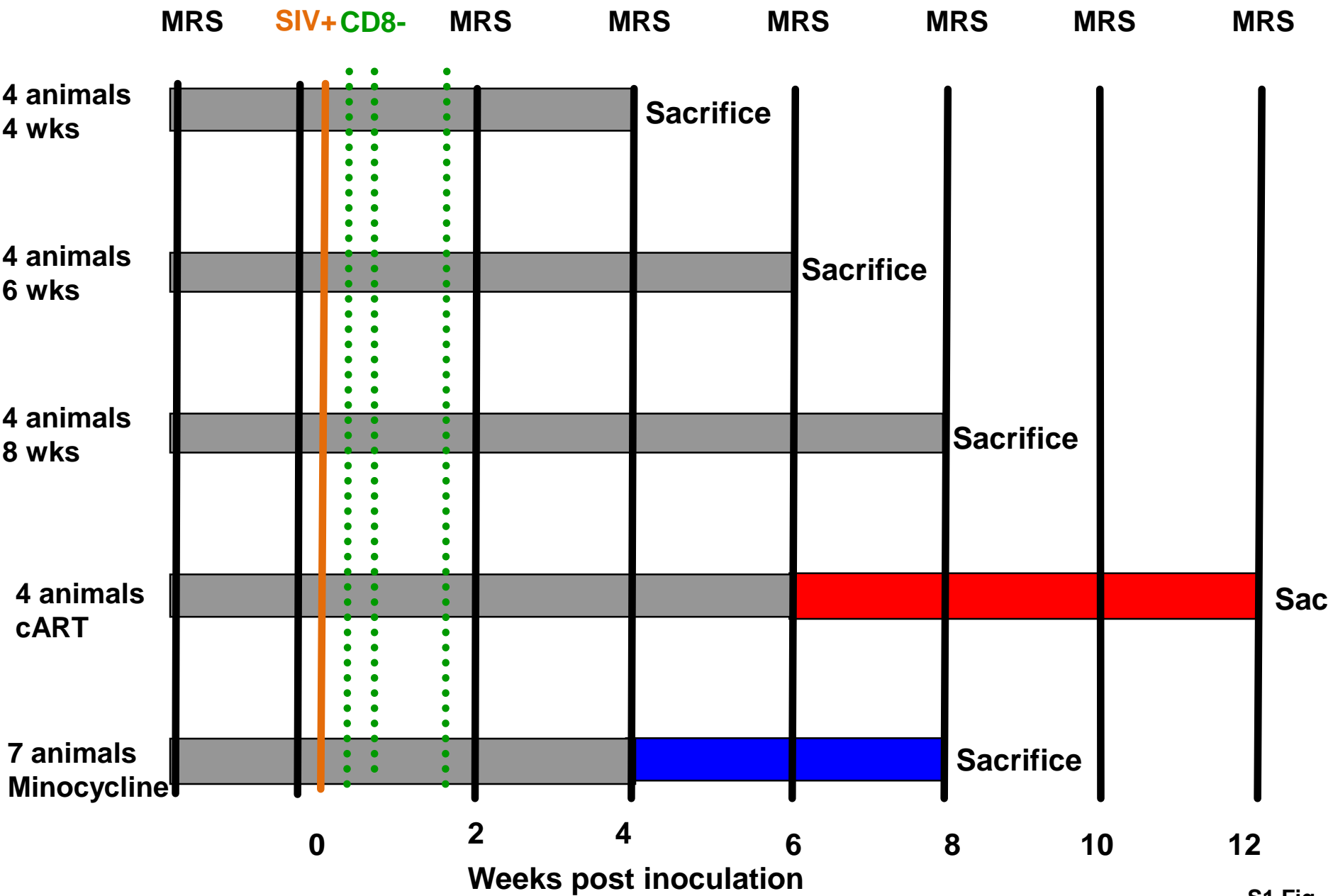

Supplement: S1 Fig — Twenty-three animals were infected with SIVmac251 virus and depleted of CD8+ T lymphocytes using anti-CD8 antibody. 12 animals remained untreated and were sacrificed at 4, 6 and 8 weeks post inoculation (wpi). Eleven animals were treated with either cART (4 animals) starting at 6 wpi or minocycline (7 animals) starting at 4 wpi. MRI and MRS was performed twice pre-inoculation and biweekly until sacrifice. (PDF) [file pone.0196949.s001.pdf]

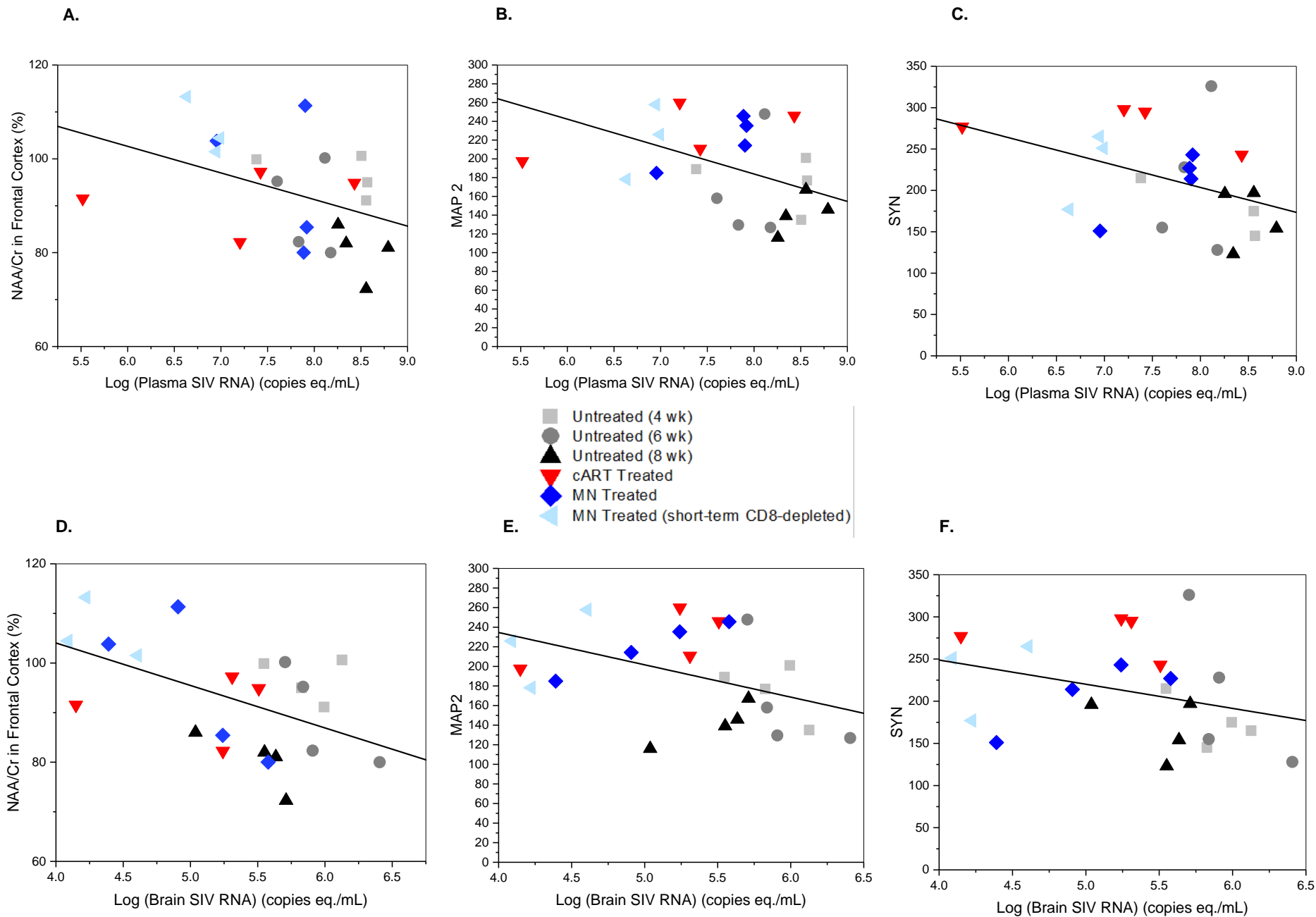

**S2 Fig.**

Supplement: S2 Fig — A. Plasma Viral Load was observed to be inversely correlated with percent changes in N-Acetylasparate/Creatine (Rρ = -0.48, P = 0.019). B. Plasma Viral Load was inversely correlated with microtubule associated protein 2 (Rρ = -0.47, P = 0.022) at endpoint. C. Plasma Viral Load was negatively correlated with synaptophysin (Rρ = -0.47, P = 0.024) at endpoint. D. Brain Viral Load was shown to be inversely correlated with percent changes in N-Acetylasparate/Creatine (Rρ = -0.48, P = 0.019) at endpoint. E. Brain Viral Load was observed to be negatively correlated with microtubule associated protein 2 (Rρ = -0.52, 0.012) at endpoint. F. Brain Viral Load was inversely correlated with synaptophysin (Rρ = -0.42, P = 0.048) at endpoint. (PDF) [file pone.0196949.s002.pdf]
